# Nano/Micro Hierarchical Bioceramic Coatings for Bone Implant Surface Treatments

**DOI:** 10.3390/ma13071548

**Published:** 2020-03-27

**Authors:** Ken-Chung Chen, Tzer-Min Lee, Nai-Wei Kuo, Cheng Liu, Chih-Ling Huang

**Affiliations:** 1 Department of Biomedical Engineering, National Cheng Kung University, Tainan 701, Taiwan; omsboy@gmail.com (K.-C.C.); tmlee@mail.ncku.edu.tw (T.-M.L.); 2 Department of Stomatology, National Cheng Kung University Hospital, Tainan 701, Taiwan; 3 Institute of Oral Medicine, College of Medicine, National Cheng Kung University, Tainan 701, Taiwan; sunny62617@gmail.com; 4 School of Dentistry, Kaohsiung Medical University, Kaohsiung 807, Taiwan; 5 Department of Dental Technology, Shu-Zen Junior College of Medicine and Management, Kaohsiung 821, Taiwan; orangeliu@gmail.com; 6 Center for Fundamental Science, Kaohsiung Medical University, Kaohsiung, 807, Taiwan

**Keywords:** nanostructures, nano-hydroxyapatite, hierarchical coatings, hydrothermal synthesis, bone implant

## Abstract

Bone implants with surface modifications that promote the physiological activities of osteoblasts are the first step for osseointegration in bone repair. Hydroxyapatite is the main inorganic component in mammal bones and teeth, and nanoscaled hydroxyapatite promotes the adhesion of osteoblastic cells. In this study, we created a nano/micro hierarchical structure using micro-arc oxidation coatings and hydrothermal treatments at 150 °C, 175 °C, and 200 °C for 2, 6, 12, and 24 h. After undergoing hydrothermal treatment for 24 h, CaTiO_3_ began forming regular-shaped crystals at the surface at 175 °C. In order to decrease the CaTiO_3_ formations and increase the apatite fabrication, a shorter time of hydrothermal treatment was required at 175 °C. There was still surface damage on samples treated for 6 h at 175 °C; however, the nano/micro hierarchical structures were formed in 2 h at 175 °C. The normalized alkaline phosphatase (ALP) activities of the MC3T3-E1 cells with micro-arc oxidation (MAO) coatings and nano/micro hierarchical bioceramics coatings were 4.51 ± 0.26 and 7.36 ± 0.51 μmol p-NP/mg protein (*** P value of <0.001), respectively. The MC3T3-E1 cells with coatings showed highly statistically significant results in terms of the ALP activity. This proposed nano/micro hierarchical structure promoted cell proliferation and osteogenic differentiation of the osteoblast MC3T3-E1 cells. This study realized a promising nano system for osseointegration via bone implant surface treatments, which can promote the physiological activities of osteoblasts.

## 1. Introduction

Injury causes fractures and disease causes osteonecrosis, and these can both result in bone defects [1]. The long treatment cycles and expensive medical costs challenge both the surgeon and the patient in the therapy process for bone defects [2]. The primary surgical treatment is bone grafting; however, the bone transplantation creates a secondary wound for the patient. Due to the natural regenerative capacity of bone tissue, there is an alternative way to cure bone defects through providing implants with osteoconduction, osteoinduction, or osteogenesis to promote bone repair. 

Titanium and its alloys are well-known for their mechanical properties, biocompatibility, and corrosion resistance in dental and orthopedic reconstructive surgery [3]. Titanium implants can be modified by various surface treatments, e.g., sandblasted and acid-etched (SLA), plasma spray, and micro-arc oxidation (MAO). SLA is a common method for surface treatment, and it can create micro and sub-micro structures on the surface [4]. Plasma spray is the most offered method to obtain a reasonable bonding strength [5]. MAO is a technique that uses a micro arc discharge to create micro-scaled ceramic coatings on Ti substrates, and it provides high hardness, high wear resistances, and supplies calcium phosphate bioactive phases for the bonding of hard tissue and implant surfaces [6]. 

Hydroxyapatite (Ca_10_(PO_4_)_6_(OH)_2_, HA) is the main inorganic component found in mammal bones and teeth. HA has attracted attention as a surface-coating compound due to its high osteoconductivity [7]. Nano HA has satisfactory osteoconductivity and osteoinductivity traits, as it is able to promote the adsorption of proteins and facilitate angiogenesis to assist with osseointegration [8]. Combining the nano and micro structures on the surface is the hierarchical topography, which mimics the complex architecture and compositions of natural bones [7].

In recent years, bone repair materials have used surface biological fixation [9,10] to enhance their applicability, including the manufacture of porous metal, which can make the bone tissue grow inward to achieve mechanical fixation [11]. A bioactive ceramic coating on the surface of the bone repair material can be used to improve the biological response, thereby forming a chemical bonding between the coating and the bone tissue to achieve fixation [12]. Ciobanu et al. [13] used a modified calcium phosphate solution containing collagen. After immersion in this solution, a bioactive ceramic coating similar to HA was deposited on the surface. This method is simple and fast at manufacturing HA coatings on a substate; however, the disadvantage is that the adhesion between the substrate and the coatings is not durable and can easily detach. 

Hydrothermal treatments can manufacture nano-scale structures. For these treatments, water is used as the solvent and the reaction is performed in a sealed pressure vessel. The hydrothermal treatment is performed under a high-pressure environment, so the reaction temperature is relatively low. The thickness, composition, and microstructure of the coating can be easily adjusted through the temperature, time, and solution. Nano HA compounds with different morphologies can be obtained through different temperatures [14]. After soaking in physiological saline for 3 months, the degradation rate of nano HA produced by the hydrothermal process is very low, and it maintains high adhesion and integrity. The recovery of damaged bone tissue usually requires 3–6 months. The chemical composition and microstructure of nano HA will affect the solubility and mineralization ability and thus affect the performance of biomedical applications [15]. 

In previous study [16], MAO-fabricated medical titanium samples were hydrothermally treated in ammonia aqueous solution (pH 11) at 200 °C for 24 and 120 h to make nanoplates or nanoleafs upon the surface to enhance bone cell adhesion, proliferation, and differentiation. It shows the feasibility of tuning morphological features of nanostructures on micro-topography, which can serve as a promising strategy to specifically modulate cellular response. However, the fabrication time required—120 h at 200 °C—is too long and energy consuming. 

In this study, we used medical titanium and the MAO technique to create coatings with a micro-structure-based surface, but nano/micro hierarchical bioceramic coatings were fabricated using a hydrothermal solution with calcium acetate and phosphate adding. Adding salts could adjust the coating composition and microstructures, and it is also speeding up the fabrication time, which is advantageous. 

Under various experimental conditions such as at 150 °C, 175 °C, and 200 °C for 2, 6, 12, and 24 h, we try to optimize the experimental parameter and create new modifications of biomaterial surfaces, which would ensure increasing the biocompatibility especially for bone cells. Furthermore, the proposed hierarchical structures were evaluated for cell activity using pre-osteoblastic cell lines (MC3T3-E1 cells). This study realized a promising surface treatment for bone implants, which can promote the cell viability of osteoblasts.

## 2. Materials and Methods 

### 2.1. Sample Preparation

A medical grade titanium disc (ASTM F67) with a 1.27 cm diameter and 0.2 cm thickness was used in this study. MAO surface pre-treatments were used for the titanium substrate. The diagram of sample preparation with the MAO system was shown in our previous study [17]. The MAO reaction device was constructed in a two-electrode electrochemical cell with a DC power supply (GPR-60H30-A, GITEK, New Taipei City, Taiwan). A stainless-steel plate was used as the cathode, and the titanium specimen was the anode. The samples were treated with an applied voltage of 350 V for 1 min at 20 °C. The electrolytes, which were composed of 0.13 M calcium acetate hydrate (Ca(CH_3_COO)_2_·H_2_O) and 0.06 M sodium phosphate monobasic monohydrate (NaH_2_PO_4_·H_2_O), were used in this study to create a calcium and phosphorus-rich deposition on the Ti surface. After the MAO treatment, the as-prepared specimens were cleaned with 95% ethanol and de-ionized water (DI water), with ultrasonic shaking (UC-D150H, DELTA, New Taipei City, Taiwan) for 5 minutes, and then, they were air dried.

**Figure 1:**
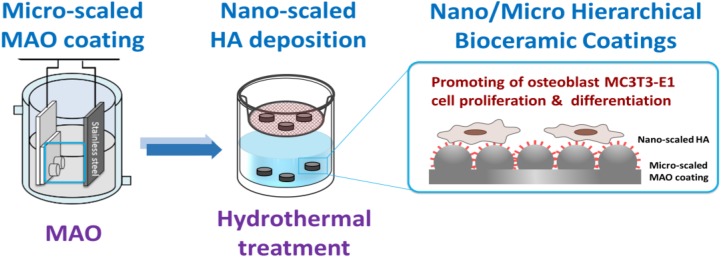
Diagram of the hierarchical structures combined with micro-scaled micro-arc oxidation (MAO) and nano-scaled hydroxyapatite (Ca_10_(PO_4_)_6_(OH)_2_, HA).

The hierarchical structures were fabricated by hydrothermal treatment, and the diagram of this is shown in Figure 1. MAO coatings were used to produce a micro-structures-based surface. According to the limitations of hydrothermal treatments, the temperature is generally controlled from 100 to 240 °C. The samples were placed in teflon capsules and placed inside an autoclave. The high temperature allows the solvent to evaporate, and the autoclave ensures that the capsules are kept in a stable system. If the temperatures were lower than 100 °C, the solvent and water were not properly evaporated. If the temperatures were higher than 260 °C, a teflon capsule inside was heated and released perfluorooctanoic acid. In order to keep the reaction system well controlled, the temperature was not higher than 200 °C in this study. 

In order to fabricate nano-scaled HA on MAO coatings, hydrothermal treatments were performed in steel autoclaves with the inner teflon capsules at 150 °C, 175 °C, and 200 °C for 2, 6, 12, and 24 h with 0.1 M NaOH, 0.02 M Ca(CH_3_COO)_2_, and 0.01 M NaH_2_PO_4_ solutions. According to a previous study, the samples can be placed in steel mesh hanging inside the teflon capsules [18] or immersed in hydrothermal solution [19]. The hydrothermal reactors had two sizes. The large one (L) had a volume of 1000 ml (containing a volume of 500 ml, 4621, Parr, Moline, IL, USA) and the ratio of containing volume to sample area (V/A ratio) was 70 ml/cm^2^. The small reactor (S) had a volume of 50 ml (containing a volume of 20 ml, Atlas, Tainan, Taiwan), and the V/A ratio was 7 ml/cm^2^. The samples were named according to their treatment conditions and listed in Table 1. 

### 2.2. Characterization of Samples

Scanning Electron Microscopy (SEM, JSM6390-LV, JEOL, Tokyo, Japan) with a LaB_6_ gun was used to observe the surface morphology and microstructures [20]. Field-emission Scanning Electron Microscopy (FE-SEM, AURIGA, Zeiss, Oberkoche, Germany) was used to observe the nano deposition on the sample surface. The chemical composition of the sample surface was analyzed with an Energy-Dispersive X-ray Spectrometer (EDS, INCA 350, Oxford Instruments, Oxfordshire, England).

Multipurpose Thin-Film X-ray Diffraction (TF-XRD, D/MAX2500, Rigaku, Tokyo, Japan) was employed to identify the crystal structure and analyze the phase orientation [21]. The scanning model was a continuous scan, and the scanning rate was 4°/sec. In this instrument, the X-ray beam was from CuKÅ radiation (λ = 1.54 Ǻ) and working at 40 kV and 100 mA. The incident beam on the surface was at an angle of 1°.

### 2.3. Cell Test in Vitro

The pre-osteoblastic cell lines (MC3T3-E1, ATCC® CRL-2593TM) that were used in this study were also used in a previous study [22]. The MC3T3-E1 cell lines are effective models for studying in vitro osteoblast differentiation, and particularly extracellular matrix signaling. They have behavior similar to primary calvarial osteoblasts. The medium of Alpha Minimum Essential Medium (α-MEM Gibco®, Thermo Fisher Scientific, Waltham, MA, USA) was used, containing 10% fetal bovine serum (FBS, Gibco®, Thermo Fisher Scientific, Waltham, MA, USA). The cells were incubated in an incubator at 37 °C with 5% CO_2_. The culture medium was changed every other day. The cells were subcultured using 0.05% trypsin-Ethylenediaminetetraacetic acid (EDTA) solution. Before the cell test in vitro, all specimens were sterilized by immersion in ethanol for 30 min. Each specimen was placed in a 24-well plate, and the cells were seeded at a number of 5000. The cell culture plates with cells were incubated in a humidified incubator containing 5% CO_2_ for 24 h for the cell adhesion test and 1, 3, and 7 days for the cell proliferation test. 

The cell viability was quantitatively assessed using a tetrazolium compound ((3-(4,5-Dimethylthiazol-2-yl)-2,5-diphenyltetrazolium bromide, MTT assay, Sigma-Aldrich Corporation, St. Louis, MO, USA) for a 4-h culture period, as according to a previous study [23]. MTT produces a colored product with a maximum absorbance at 570 nm in dimethyl sulfoxide (DMSO), and the absorbance value at 650 nm was used as a reference. The amount of colored product can be proportional to the cell number. Cells with a cell density of 5000 cells/cm^2^ were seeded on a cell culture plate and cultured at 37 °C for 24 h in a humidified incubator containing 5% CO_2_. After cultivation for 24 h, they were evaluated with an MTT assay. The data were statistically analyzed, and the results are expressed as the mean ± standard deviation.

The alkaline phosphatase (ALP) activity of the MC3T3-E1 cells was used to evaluate the osteoblast differentiation [24]. The compound p-nitrophenyl phosphate (PNPP) produces a yellow product and has maximum absorbance at 405 nm, as measured with an ELISA reader (Sunrise-basic, Tecan, Männedorf, Zürich, Switzerland). The amount of yellow product should be proportional to the MC3T3-E1 cell differentiation. 

To evaluate the statistical differences of data, we utilized a One-Way Analysis of Variance (ANOVA) technique. At least three identical samples were prepared for each experimental condition (n ≥ 3). In evaluating the test results, a * P value of <0.05 was statistically significant, a ** P value of <0.01 was very statistically significant, and a *** P value of <0.001 was highly statistically significant.

## 3. Results and Discussion

### 3.1. Characterization of the Hierarchical Bioceramics Coatings

**Figure 2:**
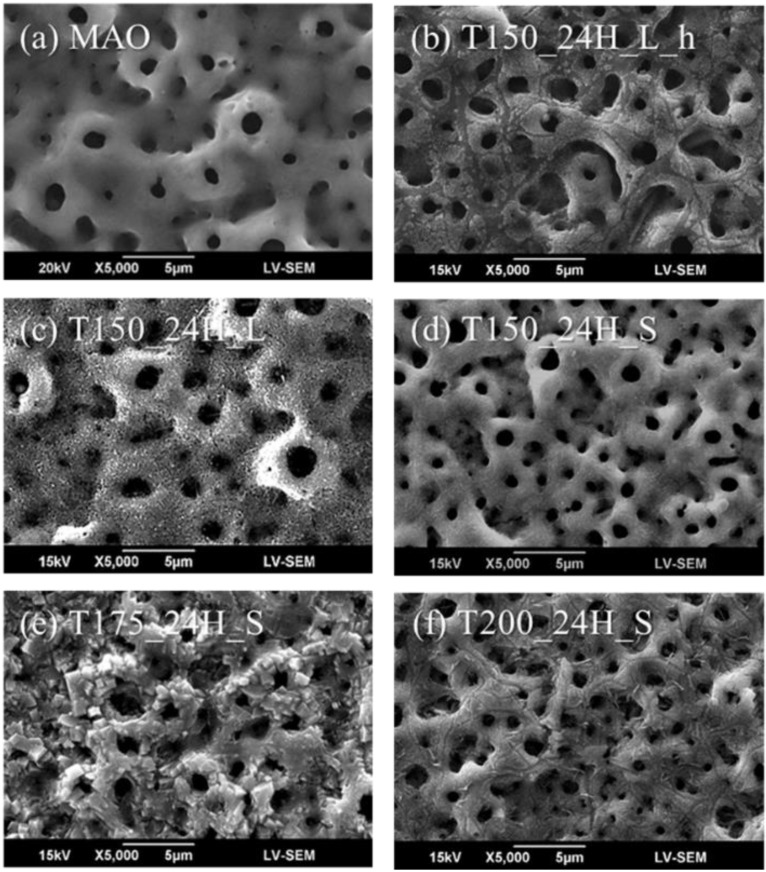
Microstructures of (**a**) the MAO coating and the hierarchical structures fabricated using hydrothermal treatment for 24 h (**b**) at 150 °C in the large reactor and placed in a hanging mesh, (**c**) at 150 °C and immersed in the large reactor, (**d**) at 150 °C and immersed in the small reactor, (**e**) at 175 °C and immersed in the small reactor, and (**f**) at 175 °C and immersed in the small reactor.

**Figure 3:**
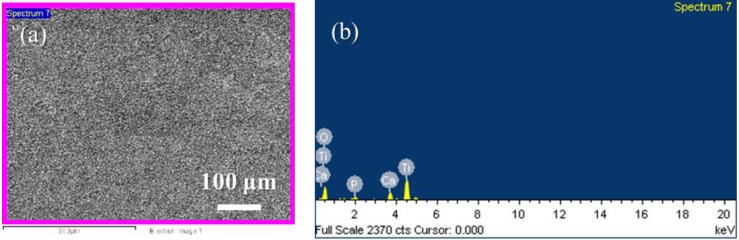
The Energy-Dispersive X-ray Spectrometer (EDS) measurement was performed on the surface of the sample. (**a**) Sample T175_24H_S was scanned in low magnification and (**b**) the EDS spectrum was collected.

MAO coatings are common implant surface treatments, and they provide the micro-scaled substrate for the next fabrication process; thus, MAO coatings were used as the control group. Figure 2 shows the microstructures, and Table 2 shows the chemical compositions of MAO and the hierarchical structures fabricated using hydrothermal treatments for 24 h at various conditions. The chemical compositions of the samples shown in Table 2 were measured by EDS. The EDS measurements were performed on a surface with low magnification to obtain more reliable average elemental compositions. For example, the highest Ca atom ratio measured by EDS was sample T175_24H_S. The EDS measurements are shown in Figure 3. In Figure 3a, the scanned area was around 330,000 µm^2^ (660 µm × 500 µm), and the region for measuring is marked in pink color. The EDS measurement was performed in low magnification to get the average results upon different regions on the sample surface. 

In Figure 2a, the MAO surface was smooth and contained many sub-micro levels with micro-pores. After hydrothermal treatment, the sub-micro levels were still present after the coatings shown in Figure 2b–f. In Figure 2b, the sample surface displayed a non-uniform coating after the hydrothermal treatment for 24 h at 150 °C in the large reactor with being placed in a hanging mesh. According to a previous study using the hanging method [18], the samples were originally deposited approximately 30 mm thick and consisted of CaHPO_4_ crystals with a plate-like morphology, with a Ca/P ratio of 1.009. However, the Ca/P ratio of MAO coatings in this study was only 0.62. This indicates that the lower amount of calcium and phosphate of samples when forming HA crystals made the value of the Ca/P ratio decrease to 0.42, due to the coatings dissolving after the hydrothermal treatment. 

In Figure 2c, uniform particles appeared upon the surface after hydrothermal treatment for 24 h at 150 °C in the large reactor and created a surface with nano-scaled roughness. In Figure 2d, there were some particles that appeared on the surface after hydrothermal treatment for 24 h at 150 °C in the small reactor and created surface roughness; however, the surface characteristics were still similar to MAO. The value of the Ca/P ratio of samples T150_24H_L and T150_24H_S were 1.11 and 0.76. This indicates that in the large reactor, the higher V/A ratio can increase the forming of HA, but the raw materials in the large reactor were 10 times the amount in the small reactor, and this resulted in a waste of raw materials. 

In Figure 2e, regular-shaped crystals appeared upon the surface after hydrothermal treatment for 24 h at 175 °C. Compared to the MAO coating surface, the amount of calcium in sample T175_24H_S was very high. The Ca atom ratio of MAO and sample T175_24H_S were 1.72% and 5.24%, respectively. This indicates that calcium salts formed on the surface after hydrothermal treatment at 175 °C for 24 h. In Figure 2f, there were some cracks on the surface after hydrothermal treatment for 24 h at 200 °C. The formula of the hydrothermal solution was a calcium and phosphate solution with 0.1 M NaOH. When the samples were immersed in this solution under hydrothermal treatment at the high temperature of 200 °C, it was equal to the hot and alkaline treatment for samples. This could produce surface damages through the caustic effect.

The hydrothermal reaction can be visualized as a water-assisted dissolution and precipitation process between water molecules and the amorphous TiO_2_. Water molecules react with the amorphous TiO_2_ to form soluble TiO_6_^2−^ octahedra, and the alkaline solution helps the reaction to increase in speed. Then, a salt containing metal ions and weak acid radicals (i.e., Ca(CH_3_COO)_2_ in hydrothermal solution) promotes the formation of different types of perovskite-type titanate. The overall reaction is described as follows [25]:

TiOx + 4H_2_O + (1 − x/2)O_2_ → Ti(OH)_6_^2−^ + 2H^+^ (1 < x < 2) (1)

Ca(CH_3_COO^−^)_2_ ↔ Ca^2+^ + 2CH_3_COO^−^ (2)

CH_3_COO^−^ + H^+^ ↔ CH_3_COOH (3)

Ti(OH)_6_^2−^ + Ca^2+^ → CaTiO_3_↓ + 3H_2_O (4)

The regular-shaped crystals on the surface of sample T175_24H_S were titanate CaTiO_3_. In order to create the nano-scaled HA on the sample surface, we considered using the hydrothermal treatment at the lower temperature. However, sample T150_24H_S was at the lower temperature, and the surface did not satisfy these goals despite using the longer time of treatment. Therefore, the shorter time of hydrothermal treatment at the middle temperature was chosen as the alternative method, and the samples were chosen as T175_2H_S, T175_6H_S, and T175_12H_S. 

**Figure 4:**
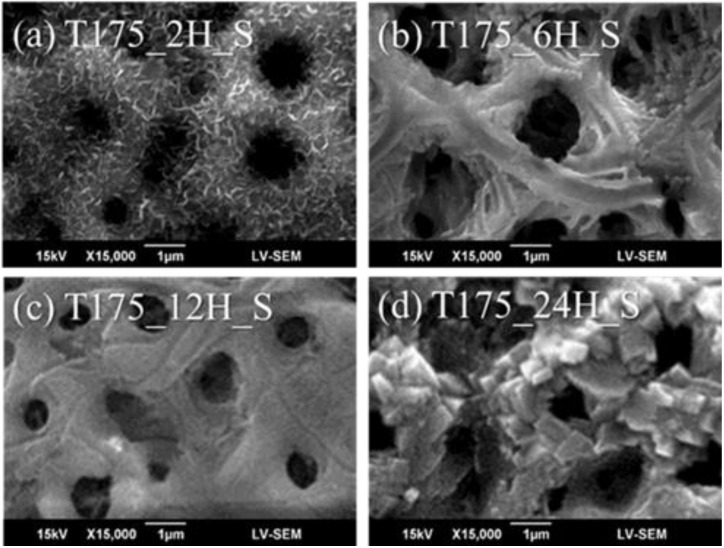
Microstructures of the hierarchical structures fabricated using a hydrothermal treatment at 175 °C for (**a**) 2, (**b**) 6, (**c**) 12, and (**d**) 24 h.

**Figure 5:**
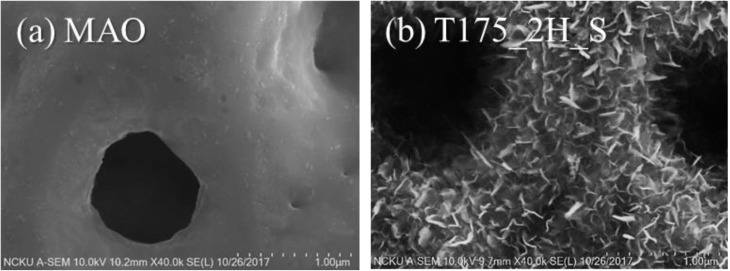
The surface morphology of (**a**) MAO coatings and (**b**) hierarchical structures fabricated using hydrothermal treatment at 175 °C for 2 h.

Figure 4 shows the microstructures of the hierarchical structures fabricated using hydrothermal treatment for 2, 6, 12, and 24 h at 175 °C. In Figure 4b,c, we still found surface damage from caustic effects with the long-time treatment. However, in Figure 3a, the hierarchical structures were fabricated using hydrothermal treatment for 2 h at 175 °C. In order to demonstrate the surface morphology combined with the nano-scaled structures to form the hierarchical character, the sample T175_2H_S, with nano-scaled depositions, which was observed by SEM, as shown in Figure 4a, was further investigated by high-resolution FE-SEM. Figure 5 shows the surface morphology of the MAO coatings and the hierarchical structures fabricated using hydrothermal treatment for 2 h at 175 °C. 

In Figure 5, the surface of the MAO coatings was smooth, but the surface of sample T175_2H_S formed a large number of nano sheets. These nano sheets were homogeneous and formed on the micro-scaled MAO coatings, even on the micro-levels of the sample surface. Moreover, the proposed coatings in this study adhered to the substrate well after the ultrasound sonication for the cleaning process. This could provide enough adhesion strength for the loading condition in clinical applications. MAO provides high hardness, high wear resistance, and supplies calcium phosphate bioactive phases for the bonding of hard tissue and implant surfaces [6]. Nano HA could maintain high adhesion and integrity after soaking in physiological saline [15]. Compared to a similar study, Huang et al. [16] used only water (pH 11) at 200 °C for 24 and 120 h to create nano/micro hierarchical structures; however, our best achievement utilized a calcium and phosphate solution at 175 °C for 2 h. This decreased the fabrication time for the hydrothermal treatment. 

**Figure 6:**
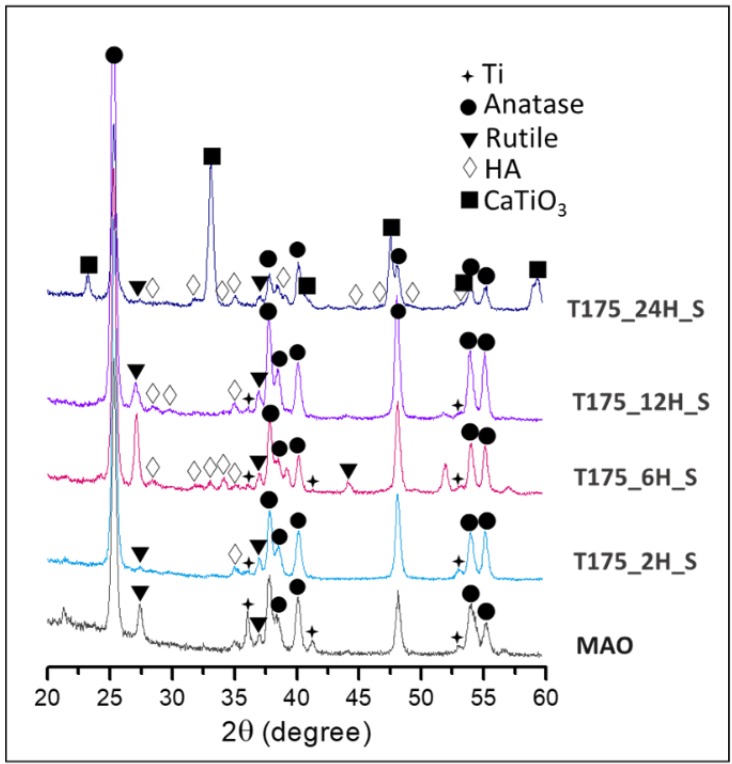
The XRD spectra of the MAO coating and the hierarchical structures fabricated using hydrothermal treatment at 175 °C for 2, 6, 12, and 24 h.

Figure 6 shows the XRD spectra of MAO and the hierarchical structures fabricated using hydrothermal treatment for 2, 6, 12, and 24 h at 175 °C. In Figure 6, there were no HA peaks for the MAO coating, as MAO was the precursor for HA forming. Crystalline diffraction peaks at 25.9°, 28.9°, 31.8°, 32.9°, 34.1°, 39.8°, 46.7°, 48.1°, and 49.5° with values of 2θ are correlated with Miller Indices of (002), (210), (211), (300), (202), (130), (222), (132), and (213), respectively, which are typical of a standard HA phase corresponding to the standard JCPDS File No. 9432. Those X-ray diffraction peaks were mainly found in sample T175_6H_S. However, these nano depositions on the MAO coating in sample T175_2H_S were ultrathin, and the peak intensities were weak. There were peaks of CaTiO3 in sample T200_24H_S, and it was strongly demonstrated that the regular-shaped crystals were titanate CaTiO3. This indicates that the calcium ions tended to form CaTiO3, but not HA, at the high temperature for 24 h. In addition, there were no peaks of CaTiO3 in sample T175_2H_S, T175_6H_S, or T175_12H_S. It was useful to decrease the CaTiO_3_ forming and increase the HA fabrication with the shorter time of hydrothermal treatment at the middle temperature of 175 °C.

### 3.2. MC3T3-E1 Cell Test in Vitro

**Figure 7:**
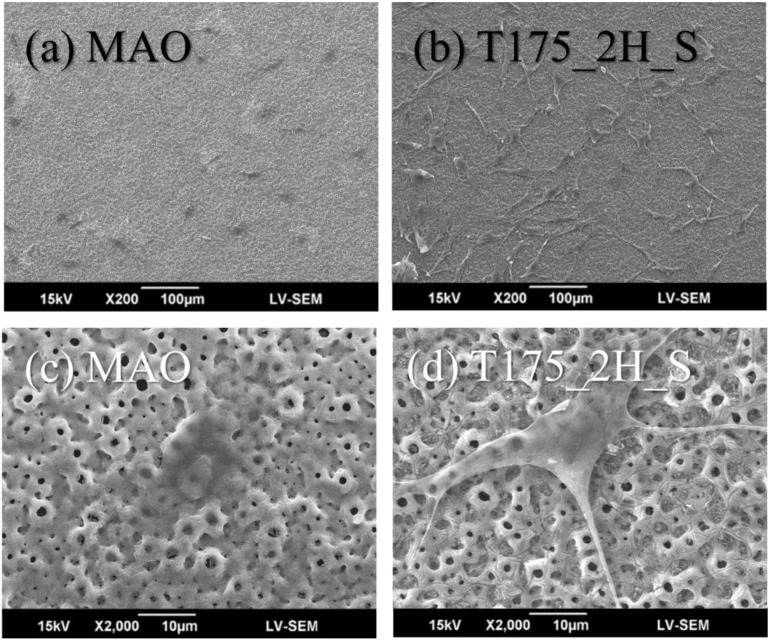
The MC3T3-E1cell morphology after 24 h of adhesion upon (**a**) MAO coatings and (**b**) the hierarchical structures fabricated using hydrothermal treatment at 175 °C for 2 h (sample T175_2H_S). (**c**,**d**) were the enlarged images of (**a**,**b**).

Figure 7 shows the MC3T3-E1 cell morphology after 24-h adhesion on various surfaces. After a 24-h adhesion, the MC3T3-E1 cells were spreading well upon the MAO coatings but tended to form a spindle-shaped pseudopodium extendng outside the cells on the hierarchical structures (i.e., sample T175_2H_S). The MC3T3-E1 cells first described by Sudo et al. in 1983, they have a fibroblastic shape, and their size were about 20 to 50 μm in diameter [26]. In Figure 7c, the dimension of MC3T3-E1 cells upon the MAO coatings were around 30–40 μm, because the cells retained a round shape and cells had no fibroblastic shape. In Figure 7d, the dimension of MC3T3-E1 cells upon the sample T175_2H_S were similar to the description in previous study. The cell size was about 20–50 μm in diameter and extended the fibroblastic filopodia. The MC3T3-E1 cell morphology was also similar to a previous study [16], with numerous filopodia formed on the nano/micro hierarchical surface. The crater-like protuberance of the microcrater surface was covered thoroughly by the fibroblastic filopodia protrusions of the cells, indicating that these structures are beneficial for cell anchorage. It is widely accepted that osteoblasts—anchorage-dependent cells—require the extracellular matrix (ECM) for anchorage to proliferate and osteogenic differentiation [27]. This indicates that the surface of hierarchical structures might enhance MC3T3-E1 cell proliferation or osteogenic differentiation [28]. In a previous study [29], the nano-HA coating had good biocompatibility and clearly suggests that the hierarchical bioceramics coatings offered a more favorable attachment for cells and lead to the formation of extracellular matrix fibrils and filopodia for osteoconductivity. 

**Figure 8:**
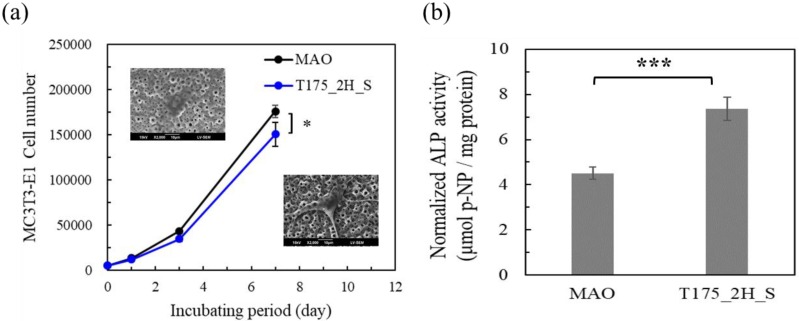
The MC3T3-E1 cells activity test in vitro on the MAO coatings and hierarchical HA coating fabricated using a hydrothermal treatment at 175 °C for 2 h (sample T175_2H_S). (**a**) The MC3T3-E1 cell number, and (**b**) normalized alkaline phosphatase (ALP) activity of the MC3T3-E1 cells.

Figure 8 shows the evaluation of the MC3T3-E1 cells activity tests in vitro. In Figure 8a, an MTT assay was used to evaluate the MC3T3-E1 cell viability after 0, 1, 3, and 7 days on different surfaces. The MC3T3-E1 cell number was evaluated after 0, 1, 3, and 7 days incubating on MAO coatings and for sample T175_2H_S. Compared to the MAO coatings, the MC3T3-E1 cell numbers both increased on these two coatings. On day 1, the MC3T3-E1 cell numbers were 13,253 ± 334 and 11,795 ± 437, for the incubations on MAO coatings and T175_2H_S, respectively. On day 3, the MC3T3-E1 cell numbers were 43,218 ± 253 and 34,396 ± 2,409, for the incubations on MAO coatings and T175_2H_S, respectively. On day 7, the MC3T3-E1 cell numbers were 175,838 ± 6659 and 150,393 ± 13,477, for the incubations on MAO coatings and T175_2H_S, respectively.

On day 7, the MC3T3-E1 cell numbers were evaluated using one-way ANOVA, and the P value of the test results was 0.042. The MC3T3-E1 cell numbers for T175_2H_S were slightly less than those for MAO; however, the growth trends both increased. The MC3T3-E1 cell numbers on day 7 for T175_2H_S increased to 30 times, and this shows the positive effect on osteoblast cell proliferation. In Figure 8b, the ALP activity of MC3T3-E1 cells was evaluated to check the osteogenic differentiation of MC3T3-E1 cells on different surfaces. 

The normalized ALP activities of the MC3T3-E1 cells on MAO coatings and hierarchical bioceramics coatings were 4.51 ± 0.26 and 7.36 ± 0.51 μmol p-NP/mg protein, respectively. The data were evaluated using one-way ANOVA, and the P value of the test results was 0.00098 (*** P value of <0.001). The ALP activity of the MC3T3-E1 cells on hierarchical bioceramics coatings was found to be highly statistically significant. Compared to a similar study [16], the cells cultured on the surface proposed in this study exhibited significantly higher ALP activity than those of the previous study. Combining the above results of the cell morphology, cell numbers, and ALP activity, the hierarchical structures show the clearly positive ability of MC3T3-E1 cells for cell proliferation and osteogenic differentiation. 

## 4. Conclusions

In this study, we successfully created hierarchical structures using hydrothermal treatments to form nano-scaled HA on micro-scaled MAO coatings. The MAO coatings contained many crater-like structures. After hydrothermal treatment, the crater-like structures still existed on the coatings. After hydrothermal treatment for 24 h, CaTiO_3_ formed regular shaped crystals on the surface at 175 °C. In order to decrease the CaTiO_3_ formation and increase the HA fabrication, a shorter time of hydrothermal treatment was required at 175 °C. There were still surface damages on the samples with 6 h at 175 °C, but the nano/micro hierarchical structures were formed after 2 h at 175 °C. The normalized ALP activities of the MC3T3-E1 cells with MAO coatings and nano/micro hierarchical bioceramics coatings were 4.51 ± 0.26 and 7.36 ± 0.51 μmol p-NP/mg protein (*** P value of <0.001), respectively. This was a highly statistically significant ALP activity for the MC3T3-E1 cells with the coatings. This proposed hierarchical structure promoted osteoblast MC3T3-E1 cell proliferation and osteogenic differentiation. This study realized a promising surface treatment for bone implants that can promote osseointegration.

## Figures and Tables

**Table 1:** Samples with various conditions for the hydrothermal process.

Samples	Temperature (°C)	Time (hour)	V/A Ratio
T150_24H_L_h *	150	24	70
T150_24H_L	150	24	70
T150_24H_S	150	24	7
T175_24H_S	175	24	7
T200_24H_S	200	24	7
T175_12H_S	175	12	7
T175_6H_S	175	6	7
T175_2H_S	175	2	7

Note: * Samples were placed in steel mesh hanging inside the teflon capsules.

**Table 2:** The chemical compositions of the MAO coatings and hierarchical structures fabricated using hydrothermal treatment for 24 h at 150 °C, 175 °C, and 200 °C.

Samples	Atomic Ratio (%)				Ca/P Ratio
	O	P	Ca	Ti	
MAO	71.14	2.78	1.72	24.36	0.62
T150_24H_L_h	72.78	1.98	0.83	24.41	0.42
T150_24H_L	74.59	1.79	1.99	21.63	1.11
T150_24H_S	71.93	0.68	0.52	26.87	0.76
T175_24H_S	71.31	1.82	5.24	21.64	2.88
T200_24H_S	72.10	0.70	1.51	25.69	2.16
